# The Respiratory Syncytial Virus Polymerase Has Multiple RNA Synthesis Activities at the Promoter

**DOI:** 10.1371/journal.ppat.1002980

**Published:** 2012-10-18

**Authors:** Sarah L. Noton, Laure R. Deflubé, Chadene Z. Tremaglio, Rachel Fearns

**Affiliations:** Department of Microbiology, Boston University School of Medicine, Boston, Massachusetts, United States of America; Centro Nacional de Biotecnologia (CSIC) and CIBER de Enfermedades Respiratorias, Spain

## Abstract

Respiratory syncytial virus (RSV) is an RNA virus in the Family *Paramyxoviridae*. Here, the activities performed by the RSV polymerase when it encounters the viral antigenomic promoter were examined. RSV RNA synthesis was reconstituted *in vitro* using recombinant, isolated polymerase and an RNA oligonucleotide template representing nucleotides 1–25 of the trailer complement (TrC) promoter. The RSV polymerase was found to have two RNA synthesis activities, initiating RNA synthesis from the +3 site on the promoter, and adding a specific sequence of nucleotides to the 3′ end of the TrC RNA using a back-priming mechanism. Examination of viral RNA isolated from RSV infected cells identified RNAs initiated at the +3 site on the TrC promoter, in addition to the expected +1 site, and showed that a significant proportion of antigenome RNAs contained specific nucleotide additions at the 3′ end, demonstrating that the observations made *in vitro* reflected events that occur during RSV infection. Analysis of the impact of the 3′ terminal extension on promoter activity indicated that it can inhibit RNA synthesis initiation. These findings indicate that RSV polymerase-promoter interactions are more complex than previously thought and suggest that there might be sophisticated mechanisms for regulating promoter activity during infection.

## Author Summary

Respiratory syncytial virus (RSV) is a major pathogen of infants with the potential to cause severe respiratory disease. RSV has an RNA genome and one approach to developing a drug against this virus is to gain a greater understanding of the mechanisms used by the viral polymerase to generate new RNA. In this study we developed a novel assay for examining how the RSV polymerase interacts with a specific promoter sequence at the end of an RNA template, and performed analysis of RSV RNA produced in infected cells to confirm the findings. Our experiments showed that the behavior of the polymerase on the promoter was surprisingly complex. We found that not only could the polymerase initiate synthesis of progeny genome RNA from an initiation site at the end of the template, but it could also generate another small RNA from a second initiation site. In addition, we showed that the polymerase could add additional RNA sequence to the template promoter, which affected its ability to initiate RNA synthesis. These findings extend our understanding of the functions of the promoter, and suggest a mechanism by which RNA synthesis from the promoter is regulated.

## Introduction

Respiratory syncytial virus (RSV) is the major cause of respiratory tract disease in infants and young children worldwide, causing 3.4 million cases of severe acute lower respiratory infection, and between 66,000 and 199,000 deaths per annum [1]. As yet, there is no vaccine available to prevent RSV disease, or effective antiviral drug to treat it [2], [3].

RSV has a single stranded, negative sense RNA genome and is classified in the Order *Mononegavirales*, Family *Paramyxoviridae*. In general terms, RSV shares the strategy for gene expression and genome replication that is used by all non-segmented negative strand (NNS) RNA viruses [4]. The RSV genome acts as a template for transcription, to generate subgenomic mRNAs, and RNA replication, to generate an antigenome RNA. The antigenome in turn acts as a template for genome RNA synthesis (reviewed in [5]). Both the genome and antigenome RNAs are encapsidated with multiple copies of nucleoprotein (N) as they are synthesized, such that each N molecule binds seven nucleotides (nts) [6]. These RNAs are never completely uncoated and so it is this N-RNA structure that acts as a template for the viral RNA dependent RNA polymerase (RdRp). To perform transcription and replication, the RdRp engages with promoter sequences that lie at the 3′ ends of the genome and antigenome RNAs [7]. The 44-nt leader (Le) promoter region at the 3′ end of the genome is responsible for directing initiation of mRNA transcription and antigenome synthesis, and the 155-nt trailer complement (TrC) promoter at the 3′ end of the antigenome directs genome RNA synthesis [8]. The organization of the Le and TrC promoters has been studied extensively using the RSV minigenome system [9]–[12]. These studies indicate that the minimal promoters are located within nts 1–11 of the genome and antigenome termini, with additional downstream sequences required for production of full-length RNA products.

Although the promoter regions of RSV and other NNS RNA viruses have been thoroughly mapped and the viral proteins involved in RNA synthesis have been identified, a detailed understanding of the molecular mechanisms underlying transcription and genome replication initiation lags significantly behind that of other RNA viruses. In part, this is due to the lack of tractable assays for studying polymerase behavior. Although the minigenome system is a valuable tool, because it is an intracellular assay it is largely limited to studying the final, stable products of these processes, and cannot be used to examine unstable RNA intermediates. It is also not possible to manipulate intracellular conditions to isolate specific steps in RNA synthesis initiation. In addition, from a drug discovery perspective, it is an expensive and time-consuming assay that is not readily applicable to a high-throughput screening approach. Study of positive strand RNA viruses has been helped enormously by the development of *in vitro* assays that reconstitute RNA synthesis using purified components e.g. [13]. Using this approach, template sequences, the polymerase and available substrates can be manipulated to perform detailed mechanistic analyses. A major hurdle to applying this approach to the NNS RNA viruses is that the natural template for their RdRp is encapsidated RNA. Although there are some reports indicating that it is possible to reconstitute N-RNA complexes *in vitro* for the rhabdoviruses [14]–[16] attempts to reconstitute RSV N-RNA complexes *in vitro* have been unsuccessful. However, available data indicate that the N protein must be locally and transiently displaced to allow the RdRp to engage the RSV RNA template in its active site [6], [17], suggesting that it might be possible to use a naked RNA oligonucleotide to recapitulate the events that occur once N protein has been locally removed from the promoter. This approach was recently applied to studying RNA synthesis initiation by the RdRp of another NNS RNA virus, vesicular stomatitis virus (VSV) [18], and now we show that it is possible to utilize this technique for studying RSV RNA synthesis initiation. Importantly, experiments with this assay, combined with analysis of RSV RNA generated in infected cells, revealed that the RSV RdRp has a far more complex behavior on its promoter than previously realized, or than has been described for VSV, with the capability of initiating RNA synthesis from two different sites on the promoter, and extending the 3′ end of the TrC RNA using a back-priming mechanism.

## Results

### Development of an *in vitro* RNA synthesis assay for the RSV RdRp

**Figure 1:**
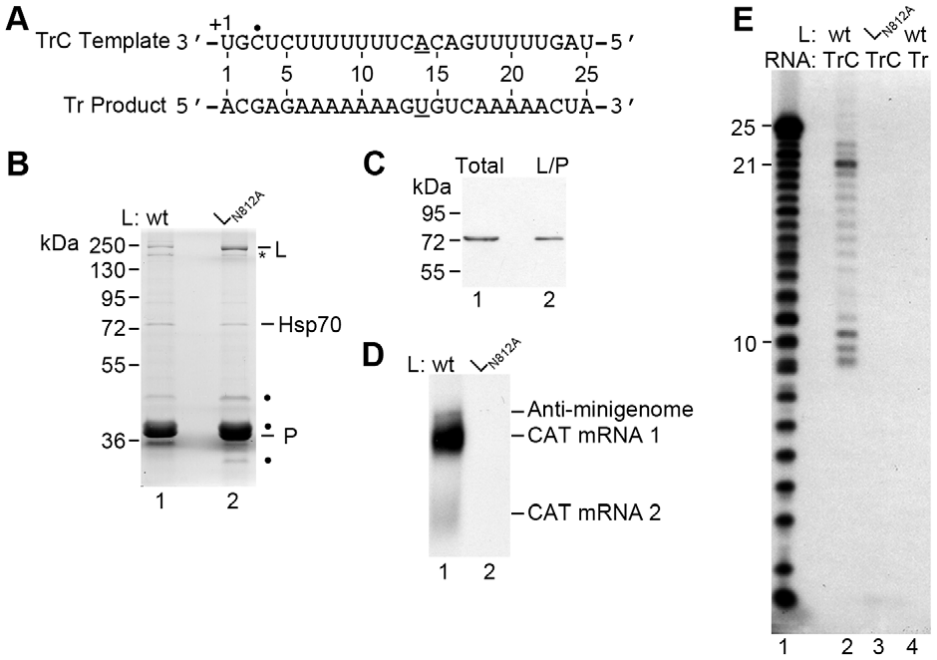
Reconstitution of RSV RNA synthesis *in vitro*. (A) Sequence of the 25 nt TrC RNA used in the *in vitro* assay and the expected complementary Tr sense product. The first A residue the RdRp would encounter at position +14 of the template and the corresponding U residue in the product are underlined. The 3′ nt of the template is marked +1, reflecting the numbering system used throughout the paper, and the +3 initiation site identified in this study is indicated by a black dot. (B) Page Blue stained gel showing isolated wt L/P (lane 1) and mutant L_N812A_/P (lane 2) complexes. The bands correlating to the expected migration patterns for L (250 kDa) and P (27 kDa) are indicated. Mass spectrometry of a representative gel showed that the bands indicated with asterisks and dots contain L and P specific polypeptides, respectively. (C) Hsp70 and/or HSC70 co-purifies with RSV L/P complexes. Total insect cell lysates (lane 1) and isolated wt L/P complexes (lane 2) were subjected to SDS-PAGE and Western blot analysis, and probed with an anti-Hsp70/HSC70 antibody. (D) Substitution of asparagine 812 to alanine in the GDNQ motif in L abolishes RSV RNA synthesis in a minigenome assay. Northern blot analysis of RSV transcription and replication products (CAT mRNAs 1/2 and anti-minigenome, respectively) generated from a dicistronic CAT minigenome in cells transfected with plasmids expressing minigenome RNA together with N, P, M2-1 and either wt L or mutant (L_N812A_), as indicated. (E) RNA products synthesized by the RSV RdRp. Isolated wt (lanes 2 and 4) or mutant (L_N812A_) (lane 3) RdRp was incubated with 0.2 µM template RNA consisting of either TrC 1–25 (lanes 2 and 3) or its complement Tr 1–25 (lane 4), with 200 µM of each NTP in the presence of [α-^32^P]ATP. The labeled products were separated by denaturing gel electrophoresis and visualized by autoradiography. Lane 1 shows the molecular weight ladder, representing nts 1–25 of the anticipated Tr product.

**Figure 2:**
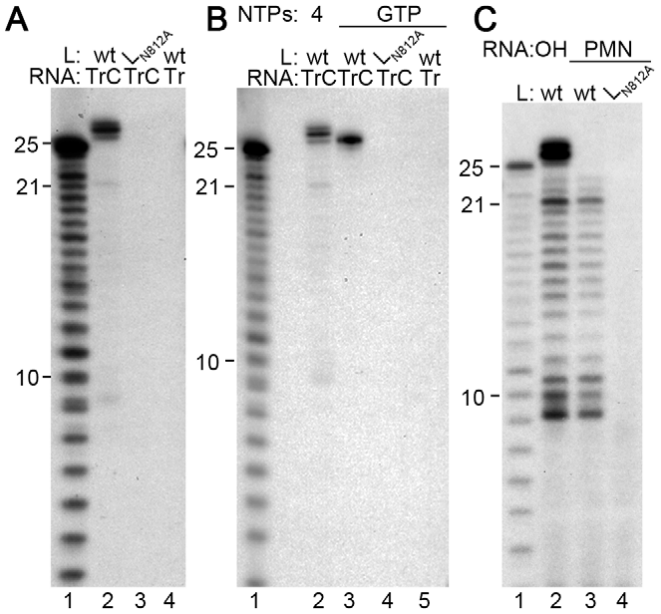
The isolated RSV RdRp adds nts to the 3′ end of the TrC template RNA. (A) A GTP label is incorporated into products of 26–28 nts in length. Wt or mutant (L_N812A_) RdRp was incubated with 0.2 µM TrC RNA template, or its complement Tr 1–25, as indicated, in a reaction containing 200 µM of each NTP and [α-^32^P]GTP. (B) GTP incorporation into the 26 nt product is independent of RNA synthesis. Reactions were performed as described for panel A, except that in lanes 3–5, the only NTP in the reaction was [α-^32^P]GTP. Lane 2 is a control containing all four NTPs and [α-^32^P]GTP. (C) Generation of the 26–28 nt products is dependent on the TrC RNA template containing a 3′-hydroxyl group. TrC RNA templates containing either a 3′-hydroxyl (OH; lane 2) or a 3′-puromycin (PMN; lanes 3 and 4) group were tested at a concentration of 2 µM in reactions containing 1 mM of each NTP and [α-^32^P]GTP. In each panel, lane 1 shows the molecular weight ladder.

To enable detailed analysis of the mechanisms involved in RSV RNA synthesis initiation, an assay was developed in which RSV RNA synthesis was reconstituted *in vitro* using isolated components. To date, the only recombinant NNS virus RdRps that have been expressed and purified in functional form are those of VSV, Chandipura and Sendai virus [18]–[23]; the purification of recombinant RdRp of RSV or any other human pathogens in the paramyxovirus family has not been described. Therefore, a strategy for purification of recombinant RSV RdRp from baculovirus infected insect cells was developed. Based on previous studies it was known that the catalytic domain for RSV RNA synthesis is located in domain III of the 250 kDa large (L) protein [24], [25], and that in infected cells, L forms a complex with the viral phosphoprotein (P), which is thought to act as a bridge between the L protein and the N protein of the nucleocapsid template [26]–[28]. Purification of the RSV L protein proved challenging for two major reasons. First, numerous attempts to express L without P were unsuccessful, indicating that whereas the VSV, Chandipura and Sendai virus L proteins can be expressed in isolation, in the case of RSV, the P protein might be necessary to stabilize L. Second, expression of L protein using the RSV gene sequence resulted in very poor expression of full-length L protein. This problem was overcome by using a codon-optimized version of the L open reading frame. By co-expressing codon-optimized L with P, it was possible to purify microgram quantities of L/P complex to near homogeneity. Figure 1B shows characteristic examples of isolated L/P complexes, with the bands corresponding to the correct migration pattern for full length L and P indicated. Note that the 27 kDa P protein has previously been shown to migrate anomalously [29], [30]. Analysis of these and other bands from a representative gel by excision, trypsin digestion and mass spectrometry, determined that the bands indicated with an asterisk or dots contained L and P specific polypeptides, respectively. The smaller L fragment may arise as a consequence of premature translation termination or proteolytic cleavage of the full length L protein. The relative abundance of this band compared to full-length L protein varied depending on the preparation. The P protein is known to be differentially phosphorylated and to exist as a highly stable oligomer [30], which could account for the multiple P bands present. The band migrating between 70 and 80 kDa was also consistently observed and identified as Hsp70 and/or HSC70 by Western blot analysis (Figure 1C). Hsp70 has previously been shown to affect RSV RdRp activity in an assay involving an infected cell extract [31], but its relevance to RSV RdRp function in the *in vitro* RNA synthesis assay described here is not yet known. Because the L/P preparations were not completely pure, and because there was variation in the relative levels of full-length and truncated L proteins, the experiments described in Figures 1 and 2 were performed with three independent preparations of wt and mutant L/P complexes and essentially identical results were obtained with each preparation.

To determine if the isolated RdRp was capable of performing RNA synthesis on a naked RNA template, L/P complexes were incubated with an RNA oligonucleotide representing the 3′ terminal 25 nts of the TrC promoter (Figure 1A) in the presence of all four NTPs and an [α-^32^P]ATP label. Although the M2-1 protein has been shown to bind P and RNA and affect transcription of mRNAs longer than ∼200 nts, it was not included in these experiments because it has been shown to have no effect on either transcription or replication initiation [32]. RNA products were analyzed by denaturing gel electrophoresis alongside a molecular weight ladder corresponding to nts 1–25 of the anticipated Tr RNA product, followed by autoradiography (Figure 1E). A number of labeled products were detected, ranging from 8 to 23 nts in length, with dominant bands of ∼8–10 nts and 21 nts (Figure 1E, lane 2). Some products longer than 25 nts could be detected at a very low level, and these are discussed in the following sections. No products were observed in reactions containing an RdRp preparation in which the L protein contained a substitution in the catalytic GDNQ motif, L_N812A_ [25] (Figure 1D; Figure 1E, lane 3), confirming that the RNA synthesis activity observed was that of the RSV RdRp. It should be noted that the bands of the molecular weight ladder do not align perfectly with the products of the RSV RdRp. For the smaller RNAs this might be in part because the RNA transcripts in the ladder contained a monophosphate group at the 5′ terminus, whereas the terminal triphosphate was removed from the products of the RSV RdRp with calf intestinal phosphatase. In addition, the ladder was designed to represent RNA initiated from +1 of the TrC promoter, but as described below, it is likely that most or all of the RSV RNA synthesis products were generated from a +3 initiation site, and so the sequences and migration patterns might not have been identical. Importantly, reactions containing an RNA template consisting of the complement of the promoter sequence (i.e. the 5′ terminal 25 nts of Tr) did not yield RNA products (Figure 1E, lane 4). This finding shows that the isolated L/P complex had RNA synthesis activity with specificity for an RNA template containing RSV promoter sequence.

### The RSV RdRp adds a specific sequence of nts to the 3′ end of the TrC RNA

**Figure 3:**
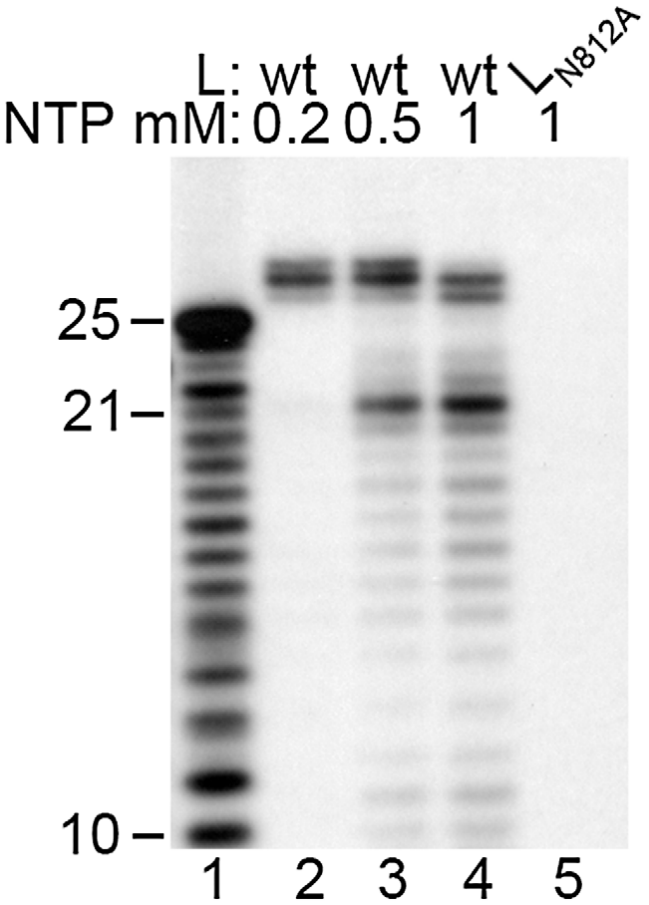
Effect of NTP concentration on RNA synthesis and 3′ nt addition. Reactions contained 0.2 µM TrC RNA with wt (lanes 2–4) or mutant (lane 5) RdRp, [α-^32^P]GTP and varying concentrations of NTPs, from 200 µM to 1 mM of each NTP, as indicated. Lane 1 shows the molecular weight ladder.

To determine if similar results were obtained with a different NTP label, reactions were performed, as described above, using [α-^32^P]GTP rather than [α-^32^P]ATP. In this case, the *in vitro* RNA synthesis reaction also resulted in products of 8–10 and 21 nts in length (Figure 2A, lane 2; note that these bands are faint in this experiment due to the relatively low NTP concentration; see Figure 3). However, dominant products of 26, 27 and 28 nts were also detected, specifically in reactions containing wt RSV RdRp and the TrC RNA. The fact that these products were larger than the input template suggested that they might have been generated as a result of the RdRp adding nts to the 3′ end of the template RNA, as has been shown for a number of other viral RdRps in *in vitro* reactions [33]–[43]. To test this possibility, reactions were performed with GTP as the only NTP source, to prevent *de novo* RNA synthesis from the TrC promoter. Under these conditions, a 26 nt band was observed (Figure 2B, lane 3). This result indicated that the 26 nt band was the result of nt addition to the 3′ end of the TrC template and was not a product of *de novo* RNA synthesis. In addition, RNA containing 3′ puromycin (PMN) in place of the 3′ hydroxyl group was tested in a reaction containing all four NTPs. The presence of 3′ PMN should abrogate 3′ terminal nt addition, while not preventing the ability of the RdRp to use the RNA as a template. The 3′ PMN TrC RNA generated significant levels of the RNAs≤23 nts, but the 26–28 nt RNA products were not detected (Figure 2C, lane 3). These results show that the RNA products smaller than 25 nts were generated by *de novo* RNA synthesis from the promoter, whereas the products longer than 25 nts were generated by addition of nts to the 3′ end of the template. In summary, the data presented in Figures 1 and 2 show that the RSV RdRp had two distinct RNA synthesis activities *in vitro*: one in which it used the TrC RNA as a template for *de novo* synthesis of RNA products, yielding a dominant product of 21 nts, minor products of 22 and 23 nts, and a series of smaller RNAs, and another in which it added additional nts to the 3′ end of the TrC RNA to generate products of 26–28 nts in length. Having identified these activities, we set out to examine the mechanisms by which they occurred.

### The relative ratios of de novo RNA synthesis and 3′ terminal extension are influenced by the NTP concentration

As a step towards optimizing the RNA synthesis assay, the NTP concentration in the reaction was varied from 200 µM to 1 mM of each NTP. At 200 µM NTP concentration, the *de novo* RNA synthesis products could be barely detected, whereas the 3′ extension products were produced at a relatively high level (Figure 3, lane 2). As the NTP concentration was increased, RNA synthesis became much more efficient (Figure 3, compare lanes 2, 3, and 4). These data show that *de novo* RNA synthesis and 3′ extension are differentially affected by NTP concentration, with *de novo* RNA synthesis depending on a higher NTP concentration than 3′ nt addition.

### Characterization of the de novo RNA synthesis products

**Figure 4:**
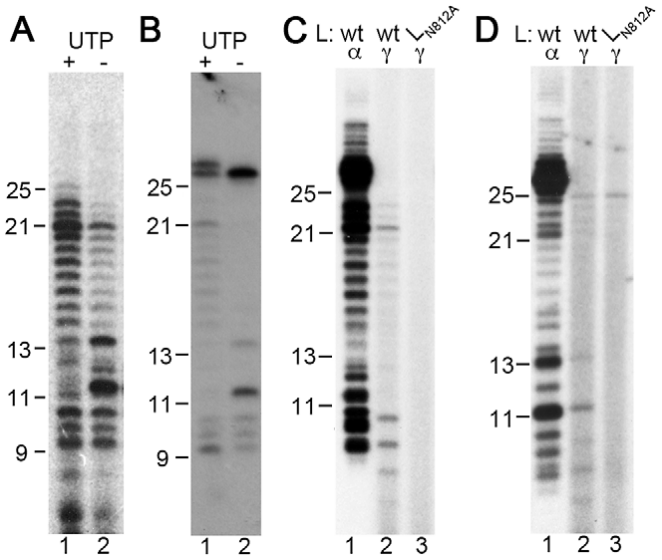
RNA products are generated from the +3 site on the TrC template. (A and B). Effect of omitting UTP from the RNA synthesis reaction. RNA synthesis reactions were performed with all four NTPs (lane 1) or with UTP omitted (lane 2). Reactions contained 2 µM TrC template RNA, wt RdRp, and 1 mM each NTP including either [α-^32^P]ATP (A), or [α-^32^P]GTP (B). (C) The 21 nt product is initiated with GTP. RNA synthesis reactions were performed with either [α-^32^P]GTP (lane 1) or [γ-^32^P]GTP (lanes 2 and 3) as a label. The reactions contained 2 µM TrC template RNA, 10 µM cold GTP and 1 mM ATP, CTP and UTP, and either wt (lanes 1 and 2) or mutant (lane 3) RdRp. (D) [γ-^32^P]GTP is incorporated into 11 and 13 nt products if UTP is omitted from the reaction. RNA synthesis reactions were performed with either [α-^32^P]GTP (lane 1) or [γ-^32^P]GTP (lanes 2 and 3) as a label. The reactions included 2 µM TrC template RNA, 50 µM cold GTP and 1 mM ATP, and CTP and either wt (lanes 1 and 2) or mutant (lane 3) RdRp. Note that the 25 nt bands in panel D, lanes 2 and 3 could be due to kinase activity (either in the RSV RdRp or a contaminant of the preparation) phosphorylating the TrC template RNA. The long products detected with [α-^32^P]GTP in lanes 1 of panels C and D might be due to extensive 3′ nt addition, or repeated stuttering of the RdRp on the U tracts in the template.

Experiments were performed to characterize the initiation and termination sites of the products of *de novo* RNA synthesis. During RSV infection, the TrC promoter directs synthesis of genome RNA, which is the full-length complement of the antigenome. Therefore, it would be expected that the RdRp would initiate RNA synthesis from the 3′ terminal nt of the TrC promoter, the +1 position, and continue RNA synthesis to the end of the template to generate a 25 nt product. The finding that the major *de novo* RNA synthesis product from the 25 nt TrC template was 21 nts in length indicated that the RSV RdRp either initiated internally and/or failed to extend to the end of the template RNA. To identify the initiation site(s), the RNA synthesis reaction was performed without UTP. As shown in Figure 1A, the first A residue in the template is at position +14, so omission of UTP should inhibit the RdRp from continuing RNA synthesis beyond nt 13. Reactions were performed with either [α-^32^P]ATP or [α-^32^P]GTP as a label (Figure 4, panels A and B, respectively). In both cases, omission of UTP resulted in a dominant band of 11 nts in length, and another band of 13 nts. However, products longer than 13 nts, including the 21 nt band, were still detectable, particularly in reactions containing [α-^32^P]ATP (Figure 4A and B, lane 2; note that there are more A than G residues in the Tr product which greatly increases the sensitivity of the [α-^32^P]ATP label). The presence of these bands suggested that either the NTP stocks were impure, or that the RdRp had poor fidelity in this assay, allowing it to insert an alternative NTP instead of UTP. Products less than 11 nts in length could also be detected, but their abundance was not affected by the presence or absence of UTP, indicating that these were premature termination products, rather than RNA initiated from downstream sites (Figure 4A and B, compare lanes 1 and 2). The fact that the 11 nt product was dominant specifically in reactions lacking UTP indicated that the RSV RdRp could initiate RNA synthesis opposite the position +3 of the TrC template. On the other hand, the 13 nt product could either be RNA that was initiated at +1 and terminated at the first A in the template at position +14, or RNA initiated at +3 and extended to the second A in the template at position +16, due to misincorporation of an NTP opposite position +14.

Therefore, as a second step to identify the initiation sites of the 11 and 13 nt products, reactions were performed using [γ-^32^P]ATP or [γ-^32^P]GTP as a label. A [γ-^32^P]NTP label can only be incorporated into the 5′ terminal nt of the product. Thus, it would be expected that RNA initiated at +1 would incorporate [γ-^32^P]ATP, whereas RNA initiated at +3 would incorporate [γ-^32^P]GTP. Despite multiple experiments with different NTP concentrations, it was not possible to clearly detect incorporation of [γ-^32^P]ATP into RNA synthesis products (data not shown). In contrast, RNA products labeled with [γ-^32^P]GTP were readily detected. In reactions lacking UTP, a product of 11 nts could be detected (Figure 4D, lane 2), providing confirmatory evidence that the RdRp could initiate opposite the C residue at position +3. A 13 nt band could also be detected (Figure 4D, lane 2). This suggested that the 13 nt RNA was generated if the RdRp initiated at position +3 and then terminated when it reached position +16. These data indicate that under these *in vitro* assay conditions, the majority of detectable RNA transcripts were initiated at nt +3.

Having identified that RNA was initiated at position +3, it was possible to deduce how far it could be extended. In reactions containing a [γ-^32^P]GTP label and all four NTPs, a product of 21 nts was generated, although smaller amounts of 22 and 23 nt products could also be detected (Figure 4C, lane 2). This indicated that the RdRp frequently paused or terminated at nt 23, with less frequent extension to the end of the template.

In summary, the data from these experiments show that during *de novo* RNA synthesis, the RdRp initiated from +3, and that while initiation at +1 might have occurred, RNA initiated from this site was not readily detectable. The data also show that the RdRp tended to pause or terminate at nt 23. In addition, the data suggest that the RSV RdRp had low fidelity under these assay conditions.

### RNA is initiated from the +3 site in RSV infected cells

**Figure 5:**
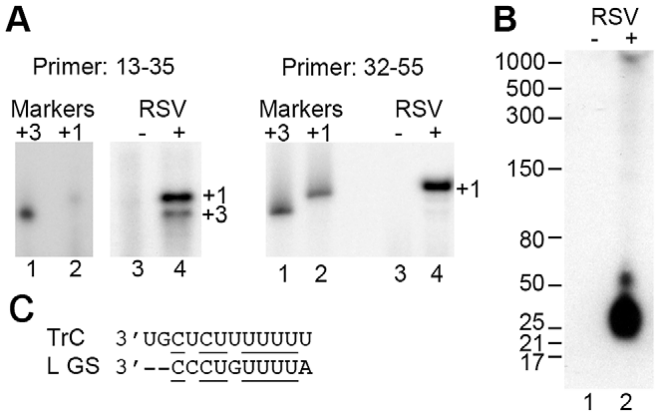
The +3 initiation site is utilized during RSV infection. (A) Primer extension analysis of Tr sense RNA generated during RSV infection. Two primers were utilized hybridizing to positions 13–35 or 32–55 relative to the 5′ terminus of RSV genome RNA (left and right panels, respectively). Lanes 3 and 4 show cDNAs generated from RNA isolated from mock or RSV infected cells, respectively. The sizes of the products were determined by co-migration of ^32^P end-labeled DNA oligonucleotides consisting of Tr sequence 3–35 or 1–35 (left panel, lanes 1 and 2, respectively), or 3–55 or 1–55 (right panel, lanes 1 and 2, respectively) to indicate the lengths of products initiated at +3 or +1. It should be noted that lanes 1–4 of the left panel are all from the same gel, but lanes 1 and 2 required a longer exposure to be detected. (B) Northern blot analysis of small genome sense RNA transcripts generated from the TrC promoter. Lanes 1 and 2 contain RNA isolated from mock or RSV infected cells, respectively. The blot was hybridized with a locked nucleic acid DNA oligonucleotide probe designed to anneal to nts 5–32 relative to the 5′ end of the RSV Tr sequence. (C) Alignment of the sequences from the 3′ terminus of the RSV TrC promoter and the ten nt L gene start (GS) signal. Identical nts are underlined and dashes indicate nts at the −1 and −2 positions relative to the L GS sequence, which are not part of the signal.

Although initiation at the +3 site of the TrC promoter has been observed previously in experiments using the RSV minigenome system [44], it has never been described during RSV infection and the size of the RNA generated from this site has not been determined precisely. Examination of the TrC sequence showed that positions +3 to +12 are almost identical to the gene start signal sequence that lies at the beginning of the RSV L gene (Figure 5C), suggesting that initiation at +3 could occur by a mechanism analogous to transcription initiation at the gene start signals that lie internally on the RSV genome. To determine if the +3 initiation site is used during infection, RNA purified from wt RSV infected cells was analyzed by primer extension using TrC-sequence specific primers. Analysis using a primer that hybridized at positions 13–35 relative to the 5′ end of the Tr sequence clearly identified two bands, corresponding to initiation at positions +1 and +3 (Figure 5A, left panel, lane 4). This finding was consistent with the results obtained with the *in vitro* RNA synthesis assay, and indicates that nt +3 is a *bona fide* initiation site. Analysis with a primer that hybridized to positions 32–55 of Tr detected RNA initiated from +1 but not from +3, indicating that whereas the RNA initiated from +1 could be elongated, the RNA generated from the +3 initiation site was not extended far enough to hybridize to this primer (Figure 5A, right panel, lane 4). To determine the size of the RNA generated from the +3 site more precisely, RNA from RSV infected cells was also analyzed by Northern blotting with a probe specific to nts 5–32 of Tr, using conditions optimized for examination of RNA of 10–500 nts in length. This analysis identified an apparently abundant RNA transcript of ∼21–25 nts (Figure 5B, lane 2). This length is consistent with the primer extension analysis of the RNA generated from the +3 site, although the data do not exclude the possibility that some of the small RNA was initiated at +1. These data show that the RSV TrC promoter has the unusual property of having two closely positioned initiation sites, one at +1 that is required to generate genome RNA, and another at +3 that yields small RNA transcripts.

### A semi-specific sequence of nts is added to the TrC 3′ terminus

**Figure 6:**
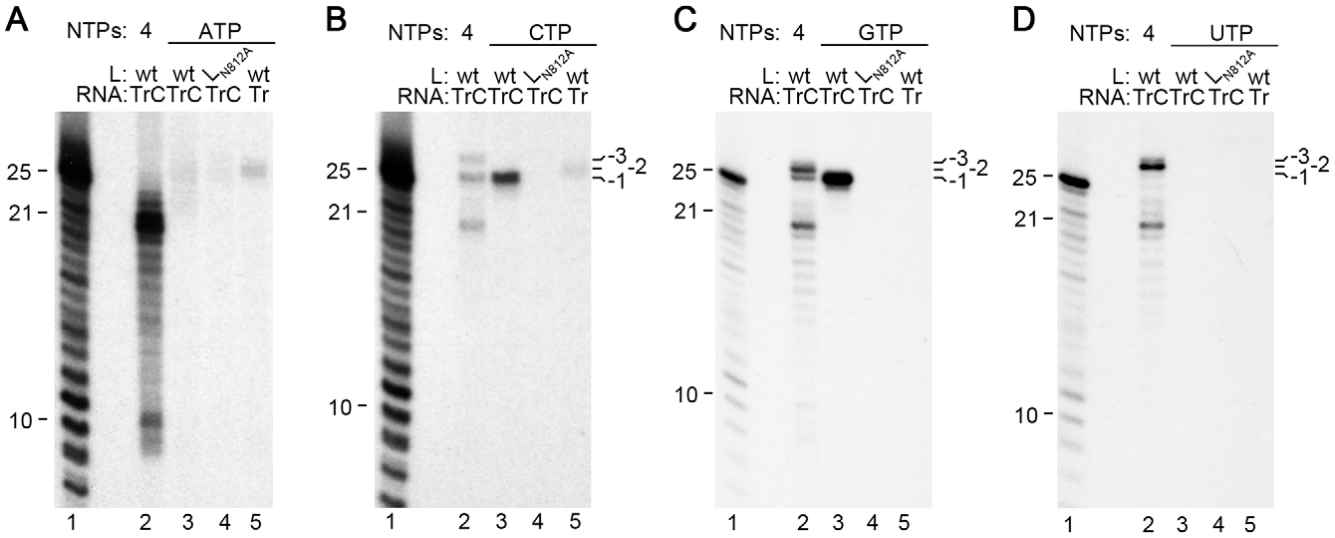
A semi-specific sequence of nts is added to the 3′ terminus of the TrC RNA. RNA synthesis reactions were performed with either [α-^32^P]ATP, [α-^32^P]CTP, [α-^32^P]GTP, or [α-^32^P]UTP (panels A–D, respectively). In each case, isolated wt or mutant (L_N812A_) RdRp was incubated with 0.2 µM TrC RNA template (lanes 2–4), or its complement Tr 1–25 (lane 5), in a reaction containing either all four NTPs (each at 500 µM; lane 2), or a single NTP (at 500 µM, lanes 3–5). Lane 1 of each panel shows the molecular weight ladder. It should be noted that to avoid confusion the marker indicators are aligned to the outermost part of the molecular weight ladder band, which in each case migrated somewhat more slowly than the rest of the gel. The position of bands representing TrC RNA containing an additional 1, 2, or 3 nts at the −1, −2, and −3 positions relative to the template, respectively, are indicated.

The data presented in Figure 2 show that in addition to generating newly synthesized RNA, the RSV RdRp could add nts to the 3′ end of the TrC RNA. Experiments were performed to determine which nts could be added, and to establish if they were added in a specific order. Reactions were performed containing each NTP label, either alone, or in combination with the other unlabeled NTPs. As described above, incubation with GTP in the absence of other NTPs showed strong incorporation into a 26 nt band, but no detectable incorporation into longer RNAs (Figure 6C, lane 3). If other NTPs were included in the reaction, a 27 nt band could be detected (Figure 6C, lane 2). This indicated that a different nt was added after the G to generate the 27 nt RNA. Labeled CTP was also incorporated into a 26 nt band in the absence of other NTPs, and yielded dominant bands of 26 and 28 nts when all four NTPs were present (Figure 6B, compare lane 3 with lane 2). In contrast, when UTP was used as a label, no incorporation was detected with UTP alone, but a 27 nt band was dominant when the other NTPs were present and a 28 nt band could be faintly detected (Figure 6D, compare lane 3 with 2). Similarly to the results shown in Figures 1 and 4, ATP showed only very weak incorporation into RNA longer than 25 nts, either in the presence or absence of other NTPs (Figure 6A). These data suggest that nts were incorporated onto the 3′ end of the TrC RNA with some specificity. Based on these data it can be deduced that either a G or C residue could be added to the −1 position at the 3′ end of the TrC RNA; a U residue could only be efficiently added after G, resulting in the 27 nt bands detected with either the GTP or UTP label, but not detectable with a CTP label; a C residue could then be added to the U to generate the 28 nt band, detected with CTP, and UTP, and to a lesser extent with a GTP label (see also Figures 2, 3 and 4). Thus, the sequence of nts most frequently added to the 3′ end of the TrC RNA was G, GU, GUC, or C only; other nt sequences, such as an A tract, might also have been added to a lesser extent. This experiment also revealed that ATP and CTP could be incorporated into the 3′ end of the Tr sense RNA also (Figure 6A and B, lane 5), but the CTP label showed that this occurred less frequently than addition to the 3′ end of the TrC RNA (Figure 6B, compare lanes 3 and 5).

### Addition of nts to the 3′ end of the TrC RNA is dependent on an internal sequence

**Figure 7:**
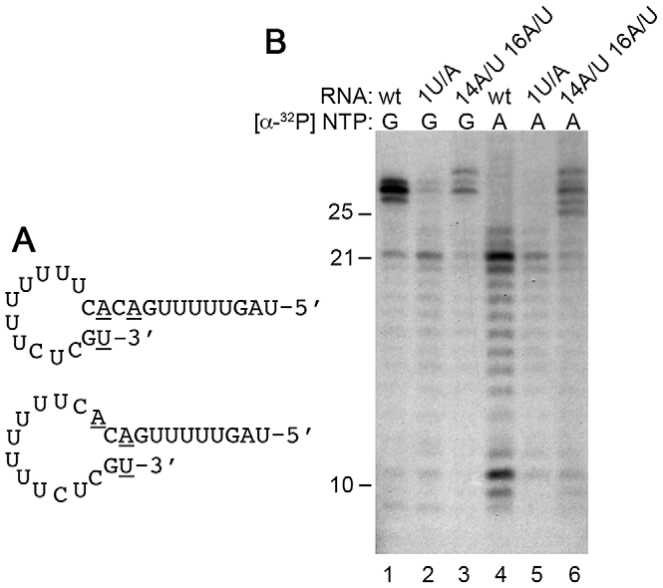
Analysis of the role of internal sequences of the TrC RNA in 3′ nt addition. (A) Schematic diagram showing the two putative hairpin loop structures formed by the TrC RNA. Nts 1, 14 and 16, which were subjected to mutagenesis are underlined. (B) Effect of mutation of nt 1, or nts 14 and 16 of the TrC RNA on 3′ nt addition. Reactions were performed containing 25 nt TrC RNA that was of wt sequence (lanes 1 and 4), or containing a 1U/A substitution (lanes 2 and 5), or substitution of nts 14A and 16A with U residues (lanes 3 and 6). Reactions were performed using 0.2 µM RNA and 500 µM of each NTP. Lanes 1–3 show RNAs labeled with [α-^32^P]GTP, and lanes 4–6 show RNAs labeled with [α-^32^P]ATP.

The mechanism by which the nts were added to the TrC RNA was investigated. There were two potential mechanisms by which 3′ nt addition could occur: terminal transferase activity, or back-priming (also known as template dependent priming). In back-priming, the 3′ end of the RNA interacts with an internal sequence to form a hairpin structure, and the RdRp adds nts to the 3′ terminus using the folded RNA as a template [38], [45]. Visual inspection of the TrC RNA sequence showed there was possibility for two alternative hairpin loop structures to form in which nt 1U could base pair with either nts 14A or 16A, and nt 2G could base pair with either nts 13C or 15C. Pairing of nts 1 and 2 with 13 and 14 and extension by one to three nts would allow the RdRp to add a G, GU, or GUC, to the 3′ end of the TrC RNA by using nts 15C-17G as a template, whereas pairing of nts 1 and 2 with 15 and 16 would allow the RdRp to add a C (Figure 7A). This model was consistent with the results shown in Figure 6. To investigate this model, nts 1 or 14 and 16 in the 25 nt TrC RNA were substituted (Figure 7A) and NTP incorporation at the 3′ end of the RNA was examined using either a GTP or ATP label. Substitution of position 1U with an A caused a significant decrease in the levels of the 26–28 nt RNAs, suggesting that the identity of the 3′ terminal nt was significant for 3′ nt addition to occur (Figure 7B, compare lanes 1 and 2). Surprisingly, substitution of positions 14A and 16A with U residues did not block 3′ nt addition, but caused an alteration in the number and sequence of incorporated nts, with A being added, and G only being incorporated into longer products (Figure 7B, compare lanes 1 and 3, and 4 and 6). Thus, disruption of possible base-pairing between the 3′ terminus and nts 13 and 14, or 15 and 16 did not prevent 3′ addition, but altered the sequence of added nts. These results show that modification of the 3′ end of the TrC RNA involves an internal sequence, consistent with a back-priming mechanism, rather than terminal transferase activity.

### A 5′-GUC-3 sequence is present at the 3′ terminus of RSV antigenome RNA

**Figure 8:**
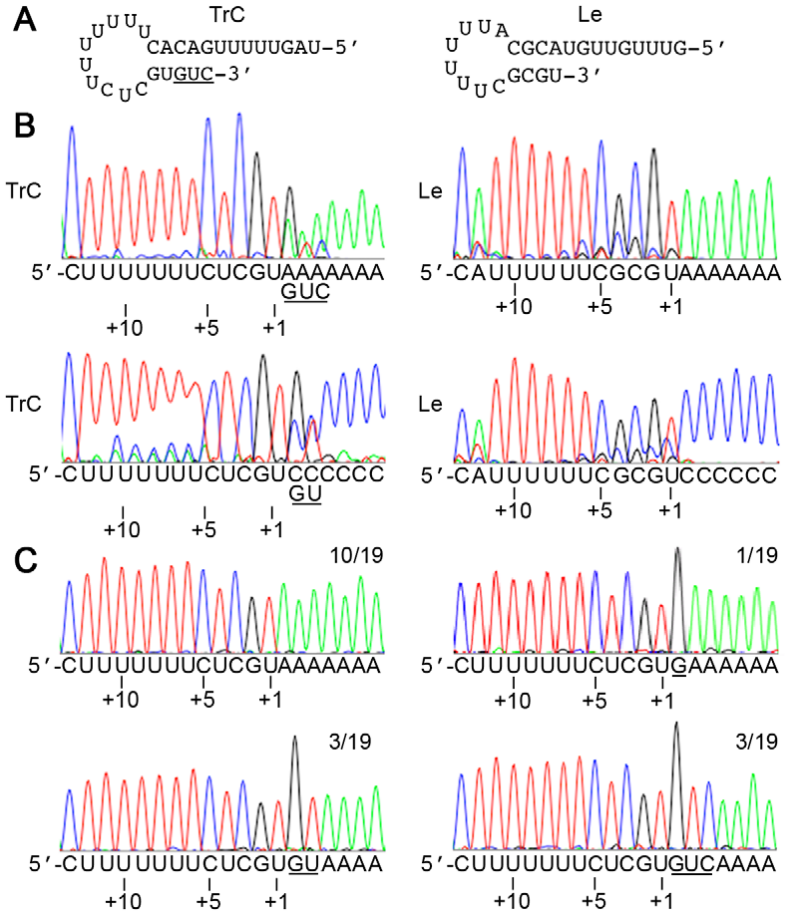
Sequence analysis of the 3′ termini of RSV antigenome and genome RNA isolated from RSV infected cells. (A) Putative structures formed by the terminal sequences of the TrC and Le promoter regions. Nts 1–25 of the TrC and Le promoter sequences are shown (left and right panels, respectively), with potential secondary structures indicated. In the case of the TrC sequence, the nts added to the 3′ end of the TrC RNA are underlined. (B) Sequence analysis of the antigenome and genome termini. The traces show the sequence of the population of cDNAs representing the antigenome and genome terminal sequences (left and right panels, respectively). In each case, the upper panel shows the sequence of RNA tailed with ATP, and the lower panel shows the sequence of RNA tailed with CTP. Note that any 3′ nt addition matching the base used to tail the RNA would not be detected. (C) Representative traces of different cDNA clone sequences obtained that represent antigenome termini. The relative frequency of each clone of the 19 clones sequenced is indicated. Two clone traces that were obtained are not shown; these contained a deletion of position 1U (or substitution with an A) with no nt additions, and the sequence 3′ CCGCGCUCUUU, in which position 1 appears to have been substituted with a C, and a GCC sequence (underlined) has been added. In panels B and C, all sequences are presented as RNA and positions +1U, +5C, and +10U of the TrC or Le promoter are indicated. The A or C residues at the right hand side of each trace represent the sequence added by the E. coli poly A polymerase, and the additional nts lying between nt +1U of the promoter and the A or C tail are underlined.

Having shown that nts were added to the 3′ end of the TrC RNA *in vitro* with some specificity, it was of interest to determine if this occurred during RSV infection. In the context of an RSV infection, the TrC sequence is at the 3′ end of the antigenome. To our knowledge, no one has previously identified additional sequences at the 3′ terminus of the RSV antigenome. However, antigenome 3′ terminal sequences are rarely determined directly, but instead are inferred from the genome sequence [7], [46]–[48]. In one paper in which antigenome RNA was analyzed, only a small number of individual clones were sequenced [49]. Thus, prior sequencing analyses did not exclude the possibility that nts are added to the TrC region of a subpopulation of antigenome RNAs during RSV infection. To examine this possibility, antigenome RNA from RSV infected cells was tailed with either A or C residues, transcribed into cDNA by 3′ rapid amplification of cDNA ends (3′ RACE) and sequenced. Direct sequence analysis of the cDNA population showed that there was a mixed population of sequences, with a significant proportion of antigenomes containing additional nts of G, U and/or C at the −1, −2, and −3 positions relative to the 3′ end of the TrC promoter, respectively (Figure 8B, left panels). Sequencing of individual cDNA clones showed that while 10/19 clones contained wt antigenome sequence with no additional nts, 7/19 clones contained a 3′ G, 3′ UG, or 3′ CUG at the end of the antigenome (Figure 8C; note that 2/19 clones did not fall into either category). These sequence additions are consistent with a back-priming event involving interaction of nts 1, 2 and 13, 14 of the TrC RNA and extension by 1–3 nts in a template dependent manner, as illustrated in Figure 8A (left panel). Examination of the Le promoter sequence at the 3′ end of the genome showed that it also has the potential to form a secondary structure that could be used to direct back-priming. Indeed, in this case, a significantly stronger secondary could be formed than by the TrC sequence (Figure 8A, right panel). However, analysis of the same RNA preparation using Le specific probes showed that there was no additional sequence at the 3′ end of the Le promoter in the genome RNA (Figure 8B, right panels), demonstrating that the 3′ end of the Le is unmodified. These findings suggest that in addition to being able to use the TrC RNA as a promoter, the RdRp also facilitates a back-priming event to allow a precise sequence of nts to be added to the 3′ end of the antigenome.

### The 3′ terminal extension inhibits RNA synthesis from the +3 site

**Figure 9:**
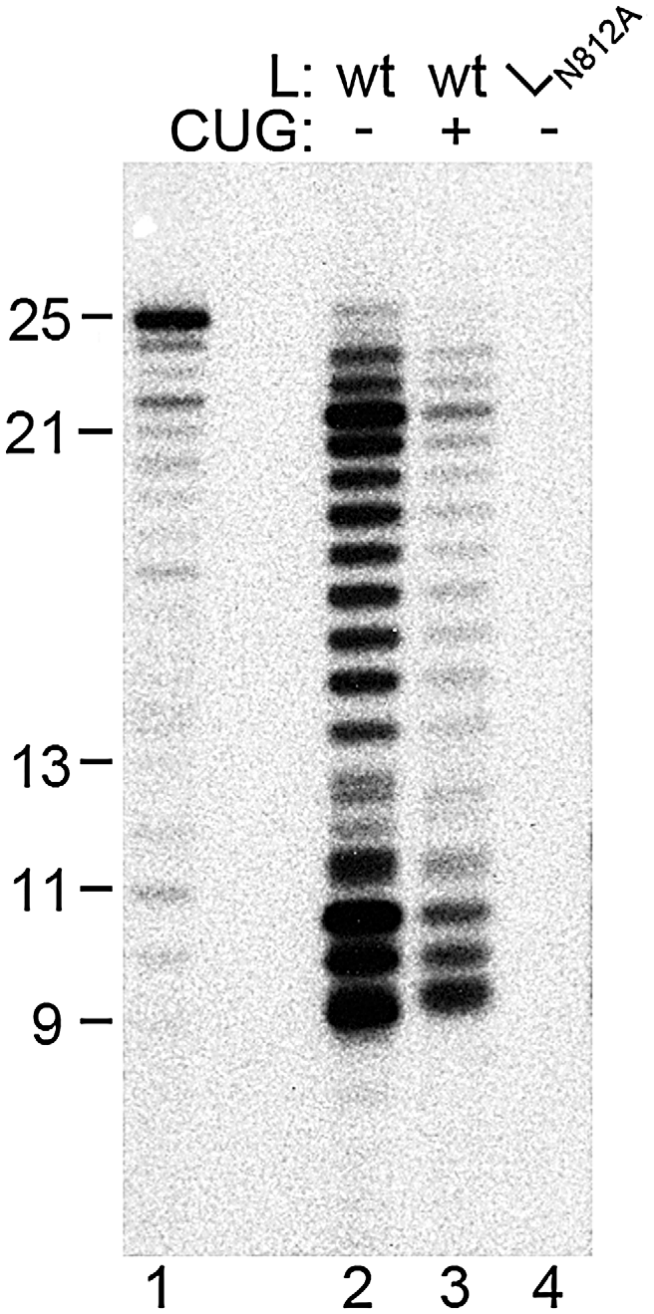
Analysis of the effect of the 3′ extension on TrC promoter activity *in vitro*. RNA synthesis reactions were performed using 2 µM of TrC template RNA either lacking (lanes 2 and 4) or containing (lane 3) a 3′ CUG addition at the 3′ terminus, 1 mM of each NTP and [α-^32^P]ATP, and wt (lanes 2 and 3) or mutant (lane 4) RdRp. Both RNA oligonucleotide templates contained a PMN group at the 3′ end. Lane 1 shows the molecular weight ladder.

The presence of additional sequence at the 3′ end of almost half of the antigenome sequences that we examined indicated that the 3′ extension plays a role in RSV replication. The only known function of antigenome RNA is as a template for RNA synthesis. Therefore, we examined if the additional nts at the 3′ end of the TrC sequence affected promoter activity. The 1–25 TrC RNA template was compared to a “+CUG” RNA template, which contained 1–25 nts of TrC sequence and a 3′ CUG extension, using the *in vitro* RNA synthesis assay. Both RNA templates contained a 3′ terminal PMN group to ensure that neither was subject to further 3′ modification. Analysis of the RNA generated from these templates showed that the presence of a 3′ terminal CUG extension was highly deleterious to RNA synthesis, indicating that the 3′ extension inhibited access of the RdRp to the promoter (Figure 9, compare lanes 2 and 3). We considered the possibility that the extension might increase initiation from the +1 position, but there was no evidence of incorporation of a [γ-^32^P]ATP label into RNA synthesized from the +CUG template (data not shown). Thus, these data indicate that the 3′ terminal extension can inhibit antigenome promoter activity.

## Discussion

This is the first report of purification of functional RSV polymerase from a recombinant source and the first time that an assay of this kind has been applied to study initiation of paramyxovirus RNA synthesis. Experiments with this assay revealed that the RSV RdRp is capable of two different RNA synthesis activities: initiation of *de novo* RNA synthesis from the TrC promoter, and 3′ extension of the RNA by a back-priming mechanism. Analysis of RNA isolated from RSV infected cells provided confirmatory evidence that there is a +3 initiation site within the TrC promoter, and the antigenome RNA can be modified by 3′ extension. These findings suggest that the interactions between the RSV RdRp and the promoter are more complex than previously thought. They also demonstrate clear differences between RSV and the prototype NNS RNA virus, VSV, but a surprising similarity with the more distantly related Borna disease virus, which has also been shown to elongate the 3′ ends of replicative RNAs [50], [51]. These results indicate that while there might be transcription and genome replication paradigms that can be applied to all the *Mononegavirales*, the details of these processes should be considered on a case-by-case basis for each virus.

### Reconstitution of RNA synthesis using a naked RNA template

It is accepted that normally the RSV template RNA is encapsidated with N protein. However, the fact that our experiments showed that the RSV polymerase was able to recognize the RNA in a sequence specific manner in the absence of N, and modify the TrC RNA apparently by using an RNA secondary structure, reveals insight into the molecular details of the polymerase-template complex. These findings suggest that although the antigenome RNA is normally encapsidated with N protein, there are occasions during the RSV replication cycle when the RdRp can interact with the RNA directly. The ability of the RSV RdRp to recognize the promoter in the absence of N protein is not necessarily surprising, as prior studies have shown that there is no requirement for the RSV promoter to be in phase with N protein [52]. Likewise, the VSV RdRp has also been shown to be able to recognize a specific initiation signal on a naked RNA template [18], and also does not follow an integer rule [53]. The situation might be different for other NNS RNA viruses, which require their genomes to be a particular integer length [54]–[60]. In these cases, the promoter is clearly recognized in the context of N protein [61]–[63] and it might be that the polymerase would either not recognize naked RNA as a template, or that there would be little or no sequence specificity on naked RNA. While RdRps are known to be error prone, the data obtained from the –UTP experiments suggest that the RdRp might have particularly low fidelity in this system (Figure 4A), which would not be tenable during infection. It is possible that during RSV infection, the N-RNA template opens to allow the RdRp to make direct contacts with the promoter RNA and initiate RNA synthesis, but that because RNA elongation is dependent on release of the RNA from the downstream N molecules, the RdRp structure is slightly altered, allowing for greater accuracy.

### Two initiation sites in the RSV TrC promoter

The TrC promoter would be expected to direct RNA synthesis initiation from the +1 position, to yield the genome RNA, and RNA initiated from +1 could be readily detected in RSV infected cells. However, RNA initiated at +3 was also detected. Primer extension analysis showed that this RNA was truncated within a short distance from the promoter and consistent with this, RNA transcripts of 21–25 nts in length could be readily detected in infected cells. The function of the small RNA initiated from +3 is not yet known. However, previous studies suggest that Tr-specific RNA might play a role in subverting the cellular stress granule response [64], [65]. If this RNA does play a functional role, it would indicate that the TrC promoter is not limited to initiating RNA replication, but also has a role in RSV transcription, albeit directing synthesis of a small RNA transcript rather than mRNA.

In this study, we showed that initiation at +3 occurs by a *de novo* initiation mechanism, and apparently does not depend on prior initiation at +1 (Figure 4). This is consistent with previous minigenome experiments that showed that mutations that inhibited initiation at +1 augmented initiation at +3 [44]. In contrast, we were unable to convincingly demonstrate initiation at the +1 position in the *in vitro* assay by incorporation of a [γ-^32^P]ATP label, despite numerous experiments aimed at optimizing NTP concentration for +1 initiation. A band of 25 nts in length could occasionally be detected (e.g. Figure 9, lane 2) indicating that a low level of initiation at +1 might occur. Failure to detect +1 initiation using [γ-^32^P]ATP could reflect differences in the NTP concentrations required for initiation at +1 versus +3. It was possible to detect +3 initiation with [γ-^32^P]GTP by using a relatively low concentration of unlabeled GTP in the reaction so that the proportion of labeled GTP in the total GTP pool was not too low. If initiation at +1 required a particularly high concentration of ATP, it might be impossible to identify conditions that allow +1 initiation, without out-competing the [γ-^32^P]ATP label with unlabeled ATP. It is also possible that initiation at +1 requires different conditions, or an additional factor that is missing in the *in vitro* assay, or that this initiation event was too inefficient to be detected with a [γ-^32^P]ATP label. In previous minigenome studies, we showed that if deletions or substitutions were introduced at position +1U of the TrC or Le promoter, almost all the detectable replication product was restored to wt sequence in a single round of replication, indicating that during initiation at the +1 site, the initiating NTP was selected independently of the 3′ terminal nt of the template [44], [66]. Based on these findings, we proposed that the RSV RdRp becomes preloaded with a primer for initiation at +1 [66]. If this model were correct, it would be expected that ATP might be required at a particularly high concentration to generate a primer and/or that other factors might also be involved. Thus, it is not surprising that the +1 initiation event could not be detected or reconstituted as readily as +3 initiation. It is unusual for an RdRp initiate from two sites within the same promoter region. One explanation for how this occurs is that the RdRp binds to a sequence within nts 3–11 of the promoter and either recruits GTP to initiate at position +3, or is preloaded with a 5′ AC or 5′ ACG primer to initiate RNA synthesis from position +1.

An *in vitro* RNA synthesis assay was also recently established for the VSV RdRp [18]. The VSV study utilized the Le, rather than the TrC promoter sequence, preventing direct comparison with the results obtained here. However, the RSV Le promoter also contains a gene start-like sequence at nts 3–12, and has been shown to direct RNA synthesis initiation from positions +1 and +3 in the minigenome system [66]. In contrast, the VSV RdRp only initiated RNA synthesis at the +1 position on the wt template, and unlike the situation with RSV, RNA initiated at this site could be readily detected using a [γ-^32^P]ATP label [18]. Thus, the existing data suggest a significant difference in the functional properties of the RSV and VSV promoters, and in their mechanisms for RNA synthesis initiation.

In the experiments shown here, there were dominant RNA synthesis products of ∼8–10 nts (e.g. Figure 1E). The reason why RNAs ∼8–10 nts in length were generated at a relatively high level is likely due to abortive synthesis in which the RdRp failed to escape from promoter and released the nascent RNA transcript. This is a common feature of initiation by RNA polymerases, which has been well documented [67]. In addition, the RdRp was inefficient at extending transcripts to the end of the template, frequently halting RNA synthesis at nt 23 of the TrC RNA (e.g. Figure 1E). Examination of RNA synthesis products from two shorter templates indicated that in one case the RdRp was able to extend to the end of the template, whereas in another it terminated either at the terminal or penultimate nt (data not shown). Therefore, it is possible that the polymerase was influenced by the 5′ terminal sequence of the template, as has been shown for two bromovirus replicases [68]. The RdRp might also terminate due to a termination signal or inherent instability at this position, as the short RNAs detected in RSV infected cells were ∼21–25 nts in length (Figure 5).

### Addition of nts to 3′ terminus of the antigenome

The data also revealed that the RSV RdRp could add nts to the 3′ terminus of the TrC RNA both *in vitro* and during RSV infection, apparently using a back-priming mechanism (Figures 2, 6, 7 and 8). These results are similar to findings for Borna disease virus, in which it has been shown that nts are added to both the genome and antigenome RNAs with an apparent 100% efficiency [51]. The finding that RSV shares a back-priming activity with Borna disease virus is surprising, as Borna disease virus is somewhat distinct from RSV and the other NNS RNA viruses. Interestingly, in the experiments described here, none of the RNA oligonucleotides tested in the *in vitro* assay possessed a stable secondary structure that would allow base-pairing between the 3′ terminus and an internal sequence, as predicted by Mfold analysis [69]. Furthermore, there was no evidence for addition of nts to the 3′ end of the RSV genome, despite the 3′ end of the Le region having the potential to form an inherently stronger RNA secondary structure (Figure 8A and B). Therefore, the RNA secondary structure to facilitate back-priming on the antigenome presumably is stabilized by the RdRp (or an associated protein) at a point when the antigenome RNA is not fully encapsidated. One possible model is that nt addition to the 3′ end of the TrC sequence occurs as the RdRp completes synthesis of the antigenome RNA. In this scenario, when the RdRp reaches the end of the antigenome, it folds the RNA into the back-priming structure and adds the additional 3′ nts before the nascent RNA becomes completely encapsidated. Alternatively, the 3′ end of the antigenome might become unencapsidated prior to RdRp binding the promoter. Then depending on RdRp orientation when it accesses the promoter, it could either modify the 3′ terminus or initiate *de novo* RNA synthesis.

It remains somewhat unclear what role the 3′ terminal extension plays in RSV replication. Unfortunately, because of the multifunctional nature of the TrC promoter in directing RNA synthesis and encapsidation, it is probably not possible to generate a mutant virus in which the 3′ terminal extension activity is ablated without also affecting other aspects of genome replication. Therefore, we can only speculate on the significance of antigenome 3′ terminal extension during RSV infection. In the case of Borna Disease virus, the function of the additional nts at the genome and antigenome ends is to compensate for cleavage of the 5′ ends of replication products, which allows the virus to avoid detection by RIG I [70]. It is unlikely that 3′ nt addition fulfills a similar function in the case of RSV, as the data suggest that only a subpopulation of replication products are modified (Figure 8). Furthermore, RIG I binds to RSV RNAs and is activated early in RSV infection [71], [72]. A second possibility is that having additional sequence and a double stranded RNA structure at the ends of the RSV RNA provides some protection of the promoter sequence from cellular exonucleases. However, if this were the case, it is not clear why there was heterogeneity in the antigenome population. We have also investigated if back-priming activity could allow repair of a template in which the 3′ terminal nt was deleted, but we were unable to detect incorporation of labeled UTP into this template in the *in vitro* RNA synthesis assay, indicating that deletion nt 1 of the TrC promoter prevents the back-priming event from occurring (data not shown). However, it is possible that repair can occur through a back-priming mechanism in a cellular environment. Finally, it is possible that the 3′ terminal additions are part of a regulatory mechanism to modulate promoter activity. Consistent with this idea, the data shown in Figure 9 indicates that the 3′ terminal extension inhibits RNA synthesis, at least in the context of the *in vitro* assay. It is important to note that in this assay the RNA was naked, and so the effect of the 3′ extension might not reflect the situation in RSV infected cells. For example, in the *in vitro* assay, the 3′ extension is likely to have created an RNA secondary structure that prevented access of the RdRp to the promoter. In contrast, in infected cells the antigenome RNA would be expected to become completely encapsidated in N protein, eliminating RNA secondary structure. Comparison of wt and mutant templates containing the extensions using the minigenome system (in which the template RNA does become encapsidated) indicated that, the 3′ terminal UG and CUG additions slightly increased RNA synthesis from both the +1 and +3 positions, while a G extension had no effect (data not shown). However, we also found that the 3′ termini of the input wt and mutant minigenome templates had the potential to be modified in the transfected cells, and so it is not clear how much weight can be attributed to these results. Nonetheless, it is interesting to speculate that the ability of the RdRp to add nts to the antigenome terminus and the effect of the nt extension are linked by the encapsidation status of the antigenome RNA. For example, one possibility is that 3′ terminal extension occurs when encapsidation of the newly synthesized antigenome RNA lags behind RNA synthesis. In this scenario, the putative hairpin structure formed by 3′ terminal extension might function to prevent initiation of *de novo* RNA synthesis until the antigenome RNA becomes fully encapsidated, at which point it might become an even more efficient promoter.

In summary, the findings presented here indicate that the behavior of the RSV RdRp at the TrC promoter sequence is more complex than previously realized, directing initiation of RNA synthesis from two sites, and having the capacity to add nts to the 3′ end of the antigenome template by a back-priming mechanism. We speculate that the advantage of having greater complexity in polymerase-promoter interactions is that it might offer an opportunity for temporal or environmental regulation of RSV RNA expression.

## Materials and Methods

### Expression and purification of the RSV L/P complex

A codon-optimized version of the RSV (strain A2) L protein ORF was chemically synthesized (GeneArt) and the mutant L_N812A_ was generated by QuickChange site-directed mutagenesis (Aligent Technologies). RSV L or L_N812A_, were cloned in the pFastBac Dual vector (Invitrogen) together with the RSV A2 P ORF, which was tagged with a hexahistidine sequence, separated from the ORF by a tobacco etch virus (TEV) protease cleavage site. Recombinant baculoviruses were recovered using the Bac-To-Bac system (Invitrogen) and used to infect Sf21 cells in suspension culture. RSV L/P protein complexes were isolated from cell lysates by affinity chromatography, TEV protease cleavage, and size exclusion. Isolated L/P complexes were analyzed by SDS-PAGE and PageBlue staining (Fermentas) and the L protein concentration was estimated against bovine serum albumin reference standards. The bands migrating between 160 to 250 kDa and ∼35 and 50 kDa were confirmed to be RSV L and P polypeptides by excising each band and performing trypsin digestion followed by liquid chromatography, tandem mass spectrometry (LC/MS/MS). The MS/MS spectra were analyzed using SEQUEST software using the RSV A2 sequence as a reference. The identity of a band migrating between 70 and 80 kDa was determined by performing SDS-PAGE and Western blot analysis using an hsp70/HSC70 specific antibody (Santa Cruz).

### Analysis of the L_N812A_ mutation using a minigenome assay

Codon optimized wt and L_N812A_ ORFs were introduced under the control of the T7 promoter in pTM1. Minigenome RNA analysis was performed using plasmid MP-28, which encodes a replication competent dicistronic CAT minigenome template [10]. Minigenome transcription and RNA replication were reconstituted in BSR-T7 cells as described previously [66]. Minigenome specific RNAs were analyzed by Northern blot using CAT-specific probes, as described previously [73].

### *In vitro* RNA synthesis

RNA oligonucleotides representing nts 1–25 of either wt or mutant TrC promoter sequence or its complement (Tr sequence) were purchased (Dharmacon). All oligonucleotides contained an –OH group at the 3′ terminus, unless stated otherwise, and an –OH group at the 5′ terminus. The RNA oligonucleotides were combined with RSV L/P (containing ∼100 ng of L protein) in transcription buffer containing 50 mM Tris HCl at pH 7.4, 50 mM NaCl, 5 mM MgCl_2_, 5 mM DTT, 40 U RNase inhibitor, and NTPs, including either 1 µl of [α-^32^P]ATP, CTP, GTP or UTP (as indicated in the legend) or [γ-^32^P]GTP (∼10 µCi), in a final volume of 50 µl. The RNA and NTP concentrations used for each experiment are indicated in the figure legends. Reactions were incubated at 30°C for 3 h, heated to 90°C for 3 min to inactivate the RdRp and cooled briefly on ice. Reactions containing [α-^32^P]NTP were combined with 10U calf intestinal alkaline phosphatase, incubated at 37°C for 1 h and RNA was isolated by phenol-chloroform extraction and ethanol precipitation. Reactions containing [γ-^32^P]GTP were combined with 7.5 µl 10% SDS, 0.5 µl 500 mM EDTA and 10 µg proteinase K and incubated at 45°C for 45 min before phenol-chloroform extraction and ethanol precipitation. The RNA was analyzed by electrophoresis on a 20% polyacrylamide gel containing 7 M urea in tris-borate-EDTA buffer, followed by autoradiography. On each autoradiogram, the nt lengths of the RNA products were determined by comparison with a molecular weight ladder generated by alkali hydrolysis of a ^32^P end-labeled RNA oligonucleotide representing the anticipated 25 nt Tr RNA product. This marker is shown in Figure 1E, Figure 2, Figure 3, Figure 6, and Figure 9, and the same marker was used for the remaining experiments. The bottom of each gel is cropped to eliminate the non-specific signal from unincorporated radiolabeled NTPs that were not always efficiently removed during RNA purification and electrophoresis.

### Primer extension and Northern blot analysis of RSV specific RNAs from infected cells

HEp-2 cells were infected with RSV at an MOI of 5 or mock infected and incubated at 37°C for 17 h. Total intracellular RNA was isolated using Trizol (Invitrogen). Primer extension reactions were carried out as described previously [44] using primers that hybridized at positions 13–35 or 32–55 relative to the genome 5′ terminus. The sizes of the labeled cDNA products were compared to ^32^P end labeled DNA oligonucleotides of sequence and length equivalent to cDNAs corresponding to RNAs initiated at positions +1 and +3 on the antigenome. The method for detecting small genome sense RNA by Northern blot analysis was adapted from a protocol described by Varallyay and co-workers [74]. Briefly, total intracellular RNA was subjected to electrophoresis in a 6% urea-acrylamide gel alongside low-range ssRNA and miRNA molecular weight standards (NEB). The lanes containing the molecular weight standards were excised from the gel prior to Northern transfer, stained with ethidium bromide and the standards were detected with UV light. The remainder of the gel was transferred to Nitran-N positively charged Nylon membrane (Sigma-Aldrich) using the Whatmann TurboBlotter downward capillary transfer system (Sigma-Aldrich) in 8 mM NaOH, 3 mM NaCl. Following transfer, blots were neutralized in 6× SSC and UV-crosslinked. Blots were prehybridized for 1 h in 5× Denhardt's Solution, 6× SSC, 0.1% SDS, and 0.01% NaPPi at and hybridized for 12–18 h with a ^32^P end-labeled locked nucleic acid modified DNA oligonucleotide specific to nts 5–32 (relative to the 5′ terminus) of genome sense RNA (5′- GAGATATTAGTTTTTGACACTTTTTTTC - 3′) in the same buffer at 62°C. Blots were washed with 6× SSC twice for 15 minutes at room temperature, and twice for 10 minutes at 62°C, and the RNA was detected by autoradiography.

### Sequence analysis of antigenome RNA in RSV infected cells

3′ RACE and sequence analysis of antigenome and genome RNA (Figure 8) was performed using RNA isolated from infected cell extracts enriched for RSV ribonucleoprotein (RNP) complexes [75]. Briefly, 8×10^6^ HEp2 cells were infected at an MOI of 5. At 17 h post infection, the supernatant was replaced with media containing 2 µg/ml actinomycin D and cells were incubated at 37°C for a further 1 h. Following an ice cold PBS wash, cells were treated for 1 min with PBS supplemented with 250 µg/ml lyso-lecithin, on ice. Cells were scraped into 400 µl of ice cold Buffer A (50 mM tris-acetate pH 8, 100 mM K-acetate, 1 mM DTT, 2 µg/ml actinomycin D), disrupted by repeated passage through an 18G needle and incubated on ice for 10 min. Following centrifugation at 2400× g for 10 min at 4°C, the resulting pellet was disrupted in 200 µl of ice cold Buffer B (10 mM tris-acetate pH 8, 10 mM K-acetate, 1.5 mM MgCl_2_, 1% triton X-100) by repeated passage through an 18G needle and then incubated on ice for 10 min. The sample was centrifuged and the resulting pellet was disrupted in 200 µl of Buffer B supplemented with 0.5% deoxycholate, 1% tween 40 as described above. Following a 10 min incubation on ice and a repeat centrifugation, the supernatant enriched for viral RNPs was collected and RNA was extracted using Trizol (Invitrogen). The purified RNA was tailed with either A or C residues using E. coli poly A polymerase (NEB), followed by heat inactivation of the enzyme, according manufacturer's instructions. First strand cDNA synthesis was performed using primers 5′ GAGGACTCGAGCTCAAGCATGCATTTTTTTTTTTTTTT, or 5′ GAGGACTCGAGCTCAAGCATGCATGGGGGGGGGGGGGGG, which hybridized to the poly A or poly C tail, respectively, and Sensiscript reverse transcriptase (Qiagen), according to manufacturer's instructions. To determine the sequence of the antigenome 3′ terminus, purified cDNA was PCR amplified using primer SLNQi 5′-GAGGACTCGAGCTCAAGC and a TrC specific primer Tr1: 5′-GCAGCACTTTTAGTGAACTAATCC. The resulting product was subjected to a second round of hemi-nested PCR using primer SLNQi and primer Tr2: 5′-GCAGTCGACCATTTTAATCTTGGAG. PCR products were gel purified and either sequenced directly or cloned into a pGEM vector for sequencing of individual cDNA clones. Analysis of the genome 3′ terminus was performed as described above, using the same cDNA preparation and primer SLNQi, but with NS1 specific primers: 5′-GCACAAACACAATGCCATTC and 5′-GCAGTCGACGTATGTATCACTGCCTTAGCC.
